# Angiomyolipoma of the Thoracic Wall: An Extremely Rare Diagnostic Challenge

**DOI:** 10.1155/2014/576970

**Published:** 2014-09-08

**Authors:** Georgios Gemenetzis, Eleni Kostidou, Kalliroi Goula, Vassilios Smyrniotis, Nikolaos Arkadopoulos

**Affiliations:** ^1^4th Department of Surgery, Medical School of Athens, Attikon University Hospital, Rimini 1, Attiki, 12462 Athens, Greece; ^2^Department of Pathology, Medical School of Athens, Attikon University Hospital, Rimini 1, Attiki, 12462 Athens, Greece

## Abstract

Extrarenal angiomyolipoma (AML) is an extremely uncommon lesion, accounting for less than 9% of all angiomyolipomas. We present a previously unreported case of a rarely located gigantic extrarenal angiomyolipoma at the posterolateral chest wall of a 35-year-old woman. Clinically, the lesion had all the characteristics of a benign tumor, being soft in palpation, painless, and growing in size in a slow rate. Histologically, the lesion consisted of convoluted thick-walled blood vessels without an elastic layer, interlacing fascicles of smooth muscle, and mature adipose tissue, features consistent with an angiomyolipoma. The mass was surgically removed, without any postoperative complications, and the patient has an uneventful postoperative course. Signs of local recurrence have not been observed. The purpose of this brief report is to point out the necessity of including angiomyolipoma in the differential diagnosis of adipose layer lesions.

## 1. Introduction

Angiomyolipoma (AML) is a clonal mesenchymal tumour with malignant potential, usually located at the kidney and most commonly found in middle-aged women. Renal angiomyolipoma is strongly associated with tuberous sclerosis complex (TSC), a group of autosomal dominant genetic disorders, and lymphangioleiomyomatosis. It is an invasive and recurring lesion, is prone to spontaneous bleeding, and consists of variable portions of interlacing fascicles of epithelioid smooth muscle, convoluted thick-walled blood vessels, and mature fat. Extrarenal manifestation of angiomyolipoma is uncommon and has been mostly reported in the liver. It is very rare and presents with male predominance, no association with TSC or lymphangioleiomyomatosis, and no signs of local recurrence. Extrarenal angiomyolipoma is also solitary and well circumscribed and is characterized by the lack of epithelioid tissue. The purpose of this report is to describe a case of angiomyolipoma in the posterior chest wall with a diameter of 22 cm, a location and size that have never been reported previously in the literature.

## 2. Case Presentation

**Figure 1:**
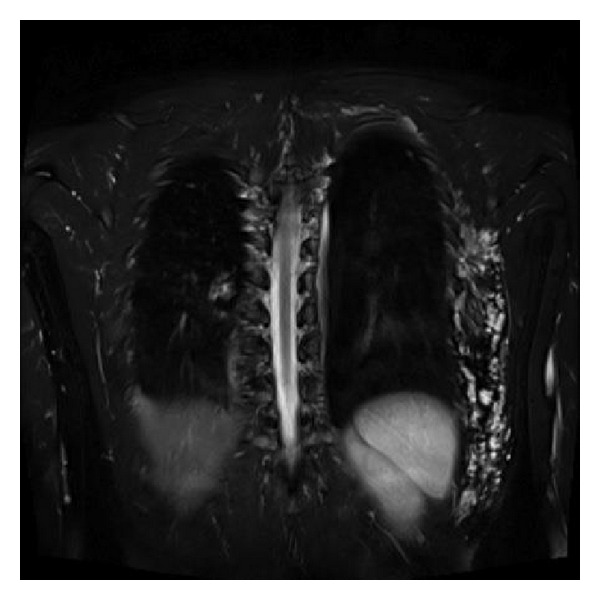
MRI scan (coronal view) of the lesion, which is located under the left scapula and extending posterolaterally down to the rim of the left 10th rib.

**Figure 2:**
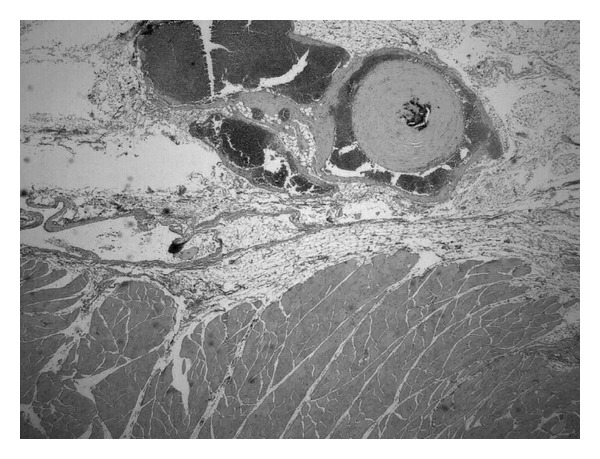
H-E staining (10x) that highlights the three distinct components of the angiomyolipoma: thick-walled blood vessels, mature adipose tissue, and interlacing smooth muscle fibers.

The patient is a 35-year-old, otherwise healthy, white female, who presented to our clinic with a large tender mass in the left posterolateral chest wall. The physical examination revealed a solitary and painful nodule adherent to deep layers and covered with normal skin. The patient's medical history included a resection of a cystic formation of the right lung in childhood. She did not receive any systemic treatment and had no family history of tuberous sclerosis complex or other inherited conditions. MRI was performed and the existence of a 22 cm lobular mass was confirmed. It was located just over the lower limit of the left scapula and extended posterolaterally roughly down to the rim of the left 10th-11th rib (Figure 1). The lesion's attributes on the MRI images advocated for a hemangioma-like lesion. The mass was surgically removed, without any postoperative complications. The specimen's histopathologic report described a mass measuring 22 × 15 cm with solid and cystic characteristics. Microscopically, a histological image of angiomyolipoma was observed. The diagnosis was based on the composition of the tumour, which consisted of convoluted thick-walled blood vessels without an elastic layer, interlacing fascicles of smooth muscle, and mature adipose tissue (Figure 2). Features of malignancy were not observed. The lesion tested negative for reaction to HMB-45 antibody, an immunohistochemical test to rule out epithelioid tumour involvement. Twelve months after surgery the patient is free of disease and the lesion has not presented any signs of local recurrence.

## 3. Discussion

Originally, angiomyolipoma was known as a hamartomatous lesion. It is now considered to be a mesenchymal tumour with malignant potential [1], usually observed in the kidney and very often (25–50%) accompanying the tuberous sclerosis complex (TSC) [2]. TSC is a group of autosomal dominant genetic disorders caused by germline mutations in the TSC1 and TSC2 genes, located on chromosomes 9q and 16p, respectively, and characterized by hamartomatous lesions in multiple organs [3]. The extrarenal manifestation of angiomyolipoma, apart from liver, is extremely rare (Table 1) and not associated with TSC, as is the case with our patient. Extrarenal angiomyolipomas usually present with clinical features similar to those of a lipoma, a hemangioma-like lesion, or an epidermal cyst, which are all included in the differential diagnosis.

MRI imaging is the most important diagnostic tool in distinguishing angiomyolipomas, since the proportion of fat in the lesion defines the ability for imaging. Macroscopic fat has high signal intensity on T1 and T2 sequence and leads to high sensitivity in the detection of extrarenal angiomyolipomas [18]. According to Brimo et al. [19] angiomyolipomas can be classified, depending on the consistence, in three major categories: regular, epithelioid with atypia, and epithelioid without atypia. Renal angiomyolipomas belong to the second or third group. The vast majority of reported extrarenal angiomyolipomas belong to the first group (except for one case reported by D'Antonio et al. [20]) and have as components mature adipose tissue, thick-walled blood vessels, and bundles of smooth muscle cells (each component forms more than 10% of the tumour). Most importantly, these lesions lack epithelioid components. Immunohistochemical features can also assist in distinguishing extrarenal angiomyolipomas. Particularly, HMB-45 antibody is positive in renal angiomyolipomas and none of the extrarenal tumors are reactive. Our lesion was also HMB-45 negative, thus consistent with these reports.

The angiomyolipoma diagnosed in our patient in the posterolateral chest wall is generally observed in middle-aged men, is noninvasive, and shows no signs of recurrence or metastasis. The size of a regular angiomyolipoma defines the symptoms, since lesions with diameter less than four cm are usually asymptomatic, whereas those with diameter bigger than four cm may cause pain or lead to bleeding. The appropriate treatment for an extrarenal angiomyolipoma is surgical resection, whereas the absence of malignancy makes any adjuvant therapy unnecessary. The posterolateral chest wall has great extent and permits the presence of a sizeable angiomyolipoma (diameter of 22 cm). Apparently, the location of the lesion plays a significant role in its size. A year after the surgical resection, our patient is healthy and has not presented any signs of local recurrence.

We presented a previously unreported case of a rarely located gigantic extrarenal angiomyolipoma of the posterolateral chest wall and pointed out that angiomyolipoma should be included in the differential diagnosis of adipose layer lesions.

## Figures and Tables

**Table 1:** Locations of extrarenal manifestations of angiomyolipomas reported in the literature.

Location	Study	Age	Gender	Max diameter (cm)
Ear and periauricular area	Argenyi et al. [4]	67	M	1
	Val-Bernal and Mira [5]	49	M	2.5
	Lee et al. [6]	32	M	1.5
	Büyükbabani et al. [7]	38	M	2.5
	Shin et al. [8]	26	F	1

Extremities	Rodriguez-Fernandez and Caro-Mancilla [9]	58	M	4
	DeBloom et al. [10]	50	F	3
	Makino et al. [11]	16	F	2.5

Nose	Büyükbabani et al. [7]	36	M	1.5

Adrenals	Godara et al. [12]	45	F	15
	Kong et al. [13]	61	M	10
	Yener and Ozcelik [14]	45	F	6
	Hu and Xi [15]	55	F	16

Torso	Morita et al. [16]	56	F	2
	Ammanagi et al. [17]	3	F	2.5
	Gemenetzis et al. (2014)	35	F	22
